# Letter from the Editor-in-Chief

**DOI:** 10.1093/ehjdh/ztab046

**Published:** 2021-06-11

**Authors:** Nico Bruining

**Affiliations:** Erasmus MC, Thoraxcenter, Department of Cardiology, Rotterdam, The Netherlands

I hope that all of you are still doing well in these troublesome times and that we can hopefully see better times soon. Being halfway through our first full year, we present you our 2nd edition of *The European Heart Journal - Digital Health*. We thank you authors, reviewers, the editorial board, and our managing editorial office, the publications team at the European Heart House of the European Society of Cardiology (ESC) and our publisher Oxford University Press (OUP) who are all so committed to make this first phase of the journal a great success. We are counting on all of you to further stimulate the growth of the journal!

As the number of submissions has increased significantly in recent months, we have invited associate professor Dr Joost Lumens [Cardiovascular Research Institute Maastricht (CARIM), Maastricht University, Maastricht, The Netherlands] to further assist the journal in the role of deputy editor, which he enthusiastically accepted. Joost’s research area is computational cardiac electromechanics and haemodynamics, and also brings a lot of general expertise in digital health, is the direct past-chair of the ESC working group e-Cardiology, core member of the ESCs Digital Health Committee and member of the ESC Congress Program Committee for Digital Health. We wish Joost every success in this new role.

To promote your work even better, we have recently added several extra features for you. Graphical abstracts draw attention to articles, especially when using social media. That’s why we now not only strongly encourage them, but made it mandatory to include a graphical summary when you receive your article back at the time of revision. In recent months, we have received quite a few articles in which the lead author has included his or her biography. That seemed attractive to us and we added this feature now as a possibility for you. Having said this, I would still like to emphasize that you also have the option to add a video-abstract and/or audio commentaries to your submissions!

This edition starts with a group of articles on remote monitoring and mobile health. Dr Krishnamoorthy presents an infrastructure to optimize the data transmission of patients with ST-segment elevation in the acute phase from the ambulance. This infrastructure, which uses smartphones and a dedicated app, promises to provide more seamless digital integration, saving valuable time. Digital education is increasing rapidly and has its advantages and disadvantages. Dr Viljoen describes the pro and cons of a mobile teaching application for electrocardiography. We all expect that remote care will grow strongly soon. Dr Nederend reports about remote monitoring and treatment of patients with heart failure. In the same context, Dr Kolk presents a quantitative evaluation of the Dutch HartWacht telemonitoring programme based on patient-reported outcomes. The latest article here is a review of wearables to track physical activity for patients with cardiovascular disease, by Dr Hammond-Haley.

The next group of papers are about digital solution to tackle COVID-19 induced problems. Dr Brown investigated whether there are differences in using telemedicine as a substitute for physical visits within distinct groups such as the elderly, lower incomes, etc. Important is the global initiative to raise awareness of hypertension by measuring blood pressure in the month of May. In South Korea, they have launched an online version of this to keep this going during the pandemic, Dr Shin reports. Finally, Dr Shah and colleagues present a feasibility study of a virtual hospitalization in which they can remotely load antiarrhythmic drugs to patients.

Interventional cardiology is home to complex medical technologies and uses many computer-based methods. A very important physiological parameter used for treatment planning is the so-called fractional flow reserve, which shows the severity of a coronary stenosis. That can be performed using many methods. One big question here is: what could be the influence of the expertise of the operator has on the results? Dr Lal reports. Structural heart disease has become important within interventional cardiology, and their minimal invasive treatment methods are very complex. The help of modern imaging systems with their continuously improved image qualities can show more and more details and have become indispensable. We can use images for creating computer models and consequently studying haemodynamics. Dr Jahangiri reports the difficulties of building such complex models and provides suggestions for improvements. The last article in the interventional group explores the use of advanced heart sound analysis being a prognostic marker for risk stratification of patients suspected of coronary artery disease.

The last group of grouped articles are about studies in which artificial intelligence (AI) has been applied. Dr Kwon investigated whether a deep learning algorithm can detect paroxysmal supraventricular tachycardia from standard 12-lead electrocardiograms (ECGs) of patients in sinus rhythm. Another significant question, addressed by Dr Chang, is whether AI can surpass board-certified physicians and current classical computer EKG analysis algorithms in detecting myocardial infarction and different heart rhythms? Cardiovascular imaging has undergone a revolution in recent decades and is growing exponentially in capabilities, quality and numbers. This puts a considerable burden to analyse all of this, perhaps AI can help us with that? Dr Chen describes an alternative method for a fully automated assessment of heart volumes based on ECG-gated computed tomography image data using a deep learning algorithm.

Rehabilitation of patients who have undergone an ischaemic heart episode is very important and many such programmes have been implemented worldwide. Most of these programmes still involve physical contact, but can eHealth-based rehabilitation become an additional option? Dr Helmark presents the results of a feasibility study entitled eMindYourHeart. In our daily life, we see QR codes in many places and they can be very helpful. They’re also more used in medicine, Dr Faggiano informs us about the current literature on QR applications in cardiology. The very last article in this edition comes from Dr Asteggiano and is a research report on the knowledge and use of eHealth in general cardiology, an important fact with which we can further shape our education regarding the applications of Digital Health in Cardiology.

I hope you enjoy this summer edition and keep submitting your Digital Health work and do not forget to visit the 3rd Digital Health Summit from 22 to 24 October, information at: https://bit.ly/3eQCd3i.

